# Influence of Co and B_*12*_ on the growth and nitrogen fixation of *Trichodesmium*

**DOI:** 10.3389/fmicb.2015.00623

**Published:** 2015-06-18

**Authors:** Irene B. Rodriguez, Tung-Yuan Ho

**Affiliations:** ^1^Research Center for Environmental Changes – Academia SinicaTaipei, Taiwan

**Keywords:** B_12_, cyanobacteria, cobalt, nitrogen fixation, trace metal limitation, *Trichodesmium*

## Abstract

We investigated the influence of varying cobalt (Co) and B_12_ concentrations to growth and nitrogen fixation of *Trichodesmium*, a major diazotroph in the tropical and subtropical oligotrophic ocean. Here we show that sufficient inorganic Co, 20 pmol L^-1^, sustains the growth of *Trichodesmium* either with or without an additional B_12_ supply. We also found that in these culture conditions, nitrogen levels fixed by *Trichodesmium* were higher in treatments with insufficient B_12_ than in treatments with higher B_12_ availability. Under limited inorganic Co availability, ranging from 0.2 to 2 pmol L^-1^, *Trichodesmium* growth was significantly compromised in cultures without B_12_. In these low Co concentrations, addition of 400 pmol L^-1^ of B_12_ supported phytoplankton growth indicating that B_12_ supply augmented for the low Co concentrations. Our study demonstrates that *Trichodesmium* has an absolute Co requirement, which is not replaceable with Zn, and that B_12_ supply alleviates stress in cases where Co is limiting. These results show that the interlocking availabilities of Co and B_12_ may influence the growth and nitrogen fixation of *Trichodesmium* in the ocean.

## Introduction

Minor nutrients including essential trace metals and vitamins play important roles in regulating phytoplankton growth and community structure in the ocean (Morel and Price, 2003; Sunda, 2012; Sañudo-Wilhelmy et al., 2014). The interaction of biologically active trace metals with various phytoplankton groups has been broadly investigated through laboratory culture studies, which have contributed to our understanding of varied roles of trace metals in shaping the phytoplankton community structure. Among the different trace metals, there is limited information regarding the cobalt (Co) requirement by phytoplankton aside from the reported Co need by the picocyanobacteria *Synechococcus* and *Prochlorococcus* (Sunda and Huntsman, 1995; Saito et al., 2002). These pioneering works paved the way for further researches that widened the understanding of Co concentrations and its role in phytoplankton growth, its distribution and speciation in the ocean, and its interaction with other trace metals (Saito and Moffett, 2002; Saito et al., 2005, 2010; Bertrand et al., 2007; Panzeca et al., 2008, 2009; Noble et al., 2012; Swanner et al., 2014). Dissolved Co concentrations can resemble a nutrient-like vertical profile and may become limiting for phytoplankton growth because both total and bioavailable concentrations in the euphotic zone are generally low (Saito and Moffett, 2001; Saito et al., 2004; Panzeca et al., 2008). For example, dissolved Co concentrations commonly occur in the range between 15 and 81 pmol L^-1^ in the North Atlantic Ocean and between 26 and 59 pmol L^-1^ in the Southern Ocean (Panzeca et al., 2008, 2009).

The major biological demand for Co is associated with cobalamin (B_12_ hereafter), an essential organic growth factor required in several biochemical processes. It is essential because B_12_ is a cofactor of several enzymes such as methylmalonyl-CoA mutase, methionine synthase, and type II ribonucleotide reductase (Martens et al., 2002; Panzeca et al., 2008; Sañudo-Wilhelmy et al., 2014). Vitamin B_12_ is a Co-containing tetrapyrrole complex synthesized by some prokaryotes, i.e., archaea and eubacteria, while majority of eukaryotes require an exogenous source (Raux et al., 2000; Warren et al., 2002; Croft et al., 2005). The dependence of most eukaryotes on external B_12_ supply in the ocean has significant effects on phytoplankton dynamics and composition. This has been demonstrated by stimulated growth of phytoplankton upon B_12_ enrichment and observed species succession from blooms involving B_12_-producing phytoplankton to auxotrophic (B_12_-requiring) species (Panzeca et al., 2008; Koch et al., 2011; Sañudo-Wilhelmy et al., 2012). Recent field studies show further proof that B_12_ can be a limiting factor for phytoplankton growth in some oceanic regions forcing phytoplankton thriving in these areas to live under a vitamin B_12_-limiting regime (Panzeca et al., 2008, 2009; Bertrand and Allen, 2012; Bertrand et al., 2012; Sañudo-Wilhelmy et al., 2012, 2014).

In this work, we set out to determine the role of B_12_ on *Trichodesmium* growth using batch cultures with replete Fe and P supply. The non-axenic strain *Trichodesmium erythraeum* was chosen for this study on Co and B_12_ interaction because it has been successfully cultured in the laboratory using YBC-II culture medium with Co and B_12_ as components (Chen et al., 1996). The presence of Co in B_12_ indicates that its synthesis and biological functions may be influenced by ambient bioavailable Co concentrations in seawater leading to a complex interaction between Co and B_12_ availabilities. Here, we studied this interaction and how it affects the growth of *Trichodesmium* and we also studied how nitrogen fixation is influenced by B_12_ availability. *Trichodesmium*, a non-heterocystous diazotroph, is a ubiquitous phytoplankton considered as a critical source of bioavailable nitrogen in nitrogen-limited regions of the tropical and sub-tropical oceans (Capone et al., 1997; Carpenter et al., 2004; Zehr and Kudela, 2011). The genome of *Trichodesmium* shows that it contains the genes required for B_12_ synthesis (Sañudo-Wilhelmy et al., 2014); however, no study pertaining to Co and B_12_ requirement of *Trichodesmium* has been reported. The results from this work provides additional information to better understand the Co and B_12_ requirements and their possible interaction in *Trichodesmium*, which may have implications in the dynamics of phytoplankton succession and nutrient cycling in the ocean.

## Materials and Methods

### Culture Conditions

Non-axenic *Trichodesmium erythraeum* IMS101, obtained from the National Center for Marine Algae and Microbiota, was grown in polycarbonate bottles with trace-metal defined medium (Ho, 2013) modified from the original YBC-II recipe (Chen et al., 1996). Before addition of desired trace metals, we determined background trace metal concentrations in seawater using an automated flow injection-ion chromatograph pretreatment system prior to element detection by high resolution inductively coupled plasma mass spectrometer (HR-ICPMS, Element XR, Thermo Scientific; Ho et al., 2010). The background total dissolved concentrations of Fe, Mn, Zn, Ni, Cu, and Co were 0.14, 0.02, 0.21, 0.04, 0.03, and 0.002 nmol L^-1^, respectively. The trace metals were then added to the culture medium to achieve these total dissolved concentrations: Fe, Ni, Mo, Mn, Cu, Zn, and Se at 400, 100, 100, 10, 10, 10, and 10 nmol L^-1^, respectively. Metal bioavailability was controlled by adding ethylenediaminetetraacetic acid (EDTA) at 20 μmol L^-1^. EDTA can maintain the bioavailable (inorganic) concentrations of trace metals in batch culture medium to be relatively constant because EDTA would bind most of the dissolved metals and regulate the dissolved bioavailable concentrations through the reversible reaction between the metal-EDTA complex and the inorganic form. For example, when bioavailable Co is consumed by the phytoplankton, the Co-EDTA complex would dissociate to keep the complexation reaction at equilibrium and thus maintain a stable inorganic Co concentration in the medium. To maintain bioavailable Co concentrations stable, the total dissolved Co concentrations need to be relatively constant in the culture medium through the experimental period too. As shown in **Table 1**, the total dissolved Co concentration added was 10 nM and the intracellular Co concentrations only ranged from 7.6 to 11.2 pM, only accounting for about 0.1% of the total concentrations added. The total initial concentration of phosphorus was 50 μmol L^-1^. These culture conditions were intended to have sufficient P and trace metal supplies in the culture medium. The other B-vitamins, thiamine, and biotin, were added at the suggested levels of 300 and 2.0 nmol L^-1^, respectively (Chen et al., 1996).

**Table 1:** Growth rates and intracellular cobalt (Co) quotas of *Trichodesmium* grown in culture media with 20 pmol L^-1^ Co′ and varying B_12_ concentrations.

B_12_ concentration (pmol L^-1^)	Specific growth rate (day^-1^)	Intracellular Co quota		Cellular Co concentration^b^ pmol L^-1^
		mmol mol^-1^ P	nmol L^-1a^	
0	0.46 ± 0.01	0.010 ± 0.001	6.6 ± 1.8	7.6 ± 1.1
40	0.49 ± 0.01	0.009 ± 0.002	4.6 ± 0.8	8.0 ± 1.3
100	0.49 ± 0.01	0.011 ± 0.001	6.5 ± 0.9	8.2 ± 0.6
400	0.49 ± 0.01	0.011 ± 0.002	7.3 ± 1.8	11.2 ± 2.3

Growth rates were evaluated while cells were in the exponential phase of the growth period. Values for the intracellular Co quotas represent mean ± 1 SD of three replicates.

^a^Intracellular Co quota per liter of cellular volume.

^b^Cellular Co concentration per liter of seawater culture medium.

The cultures were kept in 1 L bottles initially leaving a headspace volume equivalent to 150 mL. This headspace volume increased as the incubation period progressed because a 20-mL aliquot was taken from each replicate bottle for cellular volume determination, which was done every 2 days. The bottles were manually closed until finger tight and gently shaken top over bottom to allow homogenization prior to pouring out aliquots for cell counting. Cultures were kept in a temperature-controlled growth chamber fixed at 26°C under a square-wave 12:12 h light:dark regime with photon irradiance set at 600 μmol quanta m^-2^ s^-1^, the typical irradiance in the water column where *Trichodesmium* blooms have been observed (Letelier and Karl, 1998). All cultures during the acclimatization and final experiments were subjected to this temperature and light intensity. The light intensity was achieved by placing culture bottles at appropriate distances from the light source and intensity was validated by measuring light penetration PAR using a submersible radiometer (Biospherical Instruments Inc. QSL 2100). Materials used for culturing were carefully washed with 2% Micro-90^®^ solution, rinsed, soaked with 10% hydrochloric acid solution, and rinsed thoroughly with ultrapure water prepared using a Milli-Q system. All necessary procedures were carried out in a class 100 trace-metal clean laboratory.

### Effect of Co and B_12_ on the Growth of *Trichodesmium*

In this study, we varied both Co and B_12_ concentrations to study their effects on the growth of *Trichodesmium*. The Co concentrations were varied with total dissolved concentrations of 0, 0.1, 1, and 10 nmol L^-1^ in specific treatments, which would result in inorganic Co (Co′) concentrations of 0, 0.2, 2, and 20 pmol L^-1^, respectively (Westall et al., 1976). B_12_ was varied such that treatments either have 0, 40, 100, or 400 pmol L^-1^. All treatments were carried out in triplicate 1 L polycarbonate bottles. We first carried out a set of experiments where all treatments were supplied with 20 pmol L^-1^ Co′ while varying the B_12_ concentrations. For this set, we acclimatized the cells in respective B_12_ concentrations for two transfers prior to the final experiment. The cultures were transferred to fresh media with desired Co and B_12_ concentrations when cellular volume reached about 2 × 10^6^ μm^3^ mL^-1^ and while cells were in the exponential growth phase. For each transfer, 2 mL of culture was aseptically transferred to new culture medium, which resulted in initial cellular volumes within the range 5 to 8 × 10^3^ μm^3^ mL^-1^. This volume used for inoculation also resulted in 500 times dilution of carried over trace metals and B_12_ from the previous medium. We then carried out another set of experiments with low Co′ concentrations (0, 0.2, and 2 pmol L^-1^) and two B_12_ conditions (with or without 400 pmol L^-1^ B_12_). For this set of experiments, all treatments in the first transfer were inoculated using cells grown in a medium with 20 pmol L^-1^ Co′ and 400 pmol L^-1^ B_12_. For the second transfer, cells from treatments with B_12_ in the first transfer were used to inoculate corresponding Co treatments because of low cellular volumes in the treatments without B_12_.

### Phytoplankton Growth and Intracellular Metal Quota Determination

Growth of the phytoplankton was monitored by measuring total cellular volume using a Beckman Coulter Counter Multisizer 3 with a 100 μm aperture tube (Goebel et al., 2008; Ho, 2013). Coulter Counter, based on the Coulter principle widely applied for determination of size and cellular counts, was used for quantification of cellular volume to reflect growth of *Trichodesmium*. The use of the Coulter Counter has been explained in detail in a previous report (Ho, 2013). Although other parameters such as total organic carbon or fluorescence would be more robust for monitoring phytoplankton growth, we found that use of Coulter Counter is a reliable method for this purpose. The Coulter Counter offers a precision in the 1–5% range and can be used for cultures with cellular volume as low as 5 × 10^3^ μm^3^ mL^-1^, which is equivalent to presence of 1–2 trichomes (consisting of about 10–20 cells per trichome) per ml of culture medium. During the exponential phase of growth until early stationary stage, *Trichodesmium* in different treatments were present as single trichome, and we only observed formation of colonies when it reached the latter stages of the stationary phase. Thus, we made sure to transfer cells to new media while still in the exponential phase of growth to ensure comparable biomass in the inoculum. Growth rates and intracellular metal quotas for the experiments with 20 pmol L^-1^ Co′ were evaluated while cells were in the exponential growth phase. The growth rates were evaluated between days 3 and 14, where the correlation coefficient between the natural logarithm of cellular volumes and number of days of incubation were equivalent to 0.99 or better. Intracellular metal quotas were determined by harvesting cells by filtration onto acid-washed polycarbonate filters (25 mm with 5 μm pore size) during the light period of the light:dark regime. The filtered cells were washed with ultrapure water and digested using concentrated HNO_3_ prior to elemental analysis using HR-ICPMS.

### Effect of B_12_ Supply on Nitrogen Fixation

We studied the effect of B_12_ availability, when Co is sufficient (20 pmol L^-1^ Co′), on the nitrogen fixation of *Trichodesmium*. Results from this experiment yielded different nitrogen fixation rates with varying B_12_ availabilities. Thus a similar experiment, focusing only on the lowest and highest B_12_ treatments, was carried out to ascertain that the trends observed in the preceding experiment were repeatable. Nitrogen fixation rates were determined using the acetylene reduction method (Capone and Montoya, 2001) following procedures outlined elsewhere (Ho, 2013). In brief, 10 mL aliquots of the cultures (duplicate samples were prepared from each of the triplicate bottles per treatment for each time point) were transferred to 20 mL vials (Agilent). The vials were sealed using Teflon-coated caps and 2 mL air was drawn using a syringe, which was replaced by 2 mL of freshly prepared acetylene to initiate the experiment. The vials were then incubated at the same growth conditions as the original cultures. The time-point experiment was designed so that nitrogen fixation was stopped after every 2 h during the 12 h light phase. The purity of acetylene used for the estimation of nitrogen fixation rates was different during the first and validation experiments. Acetylene prepared in the laboratory by reacting calcium carbide with water was used in the first experiment while commercially available acetylene was used in the validation experiment (99.9999% purity, Kouatsu Gas Kogyo, Japan). Laboratory-prepared acetylene resulted in blank measurements that were three times higher than in commercially available acetylene. Respective blank values were subtracted from sample measurements based on the acetylene used. All acetylene reduction assays were carried out while cells were in the exponential phase of growth and when biomass was higher than 1 × 10^6^ μm^3^ mL^-1^. Estimation of dinitrogen reduction was taken from the acetylene reduction using a conversion ratio of 4:1 (Capone and Montoya, 2001), and the assumption that the Bunsen coefficient for ethylene is 0.084 (Breitbarth et al., 2004).

## Results

**FIGURE 1:**
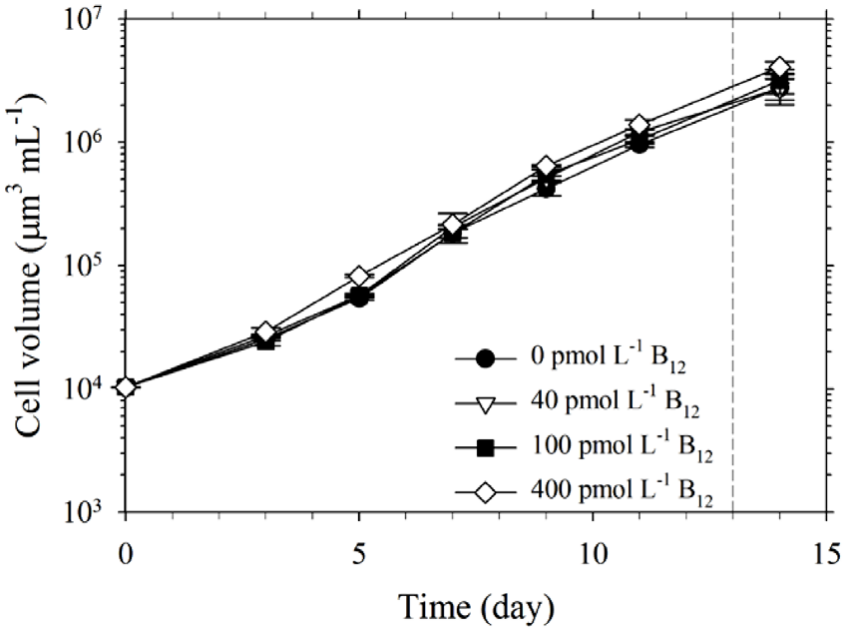
**Growth of *Trichodesmium* in culture media with 20 pmol L^-1^ Co′ and varying B_12_ concentrations.** Cells were harvested for intracellular cobalt (Co) quota determination while in exponential phase of growth (indicated by the dashed line). Values represent mean and error bars represent SD of triplicate culture bottles.

The set of treatments with 20 pmol L^-1^ Co′ yielded comparable values for growth and intracellular Co quotas. Growth rate for the treatment without B_12_ was 0.46 ± 0.01 d^-1^, while the rates determined for the treatments with 40, 100, and 400 pmol L^-1^ B_12_ were all at 0.49 ± 0.01 d^-1^. The growth rates were similar to the maximum growth rates we have obtained previously (Ho, 2013; Ho et al., 2013; Rodriguez and Ho, 2014). The comparable biomass and growth rates indicate that 20 pmol L^-1^ Co′ was sufficient for *Trichodesmium* to reach maximum growth rates and that B_12_ does not significantly affect the growth at this Co′ concentration (**Figure 1**; **Table 1**). The determined intracellular Co quotas, normalized against P as biomass indicator, were also comparable suggesting that Co uptake rates were independent of B_12_ concentrations when Co supply was sufficient (**Table 1**).

**FIGURE 2:**
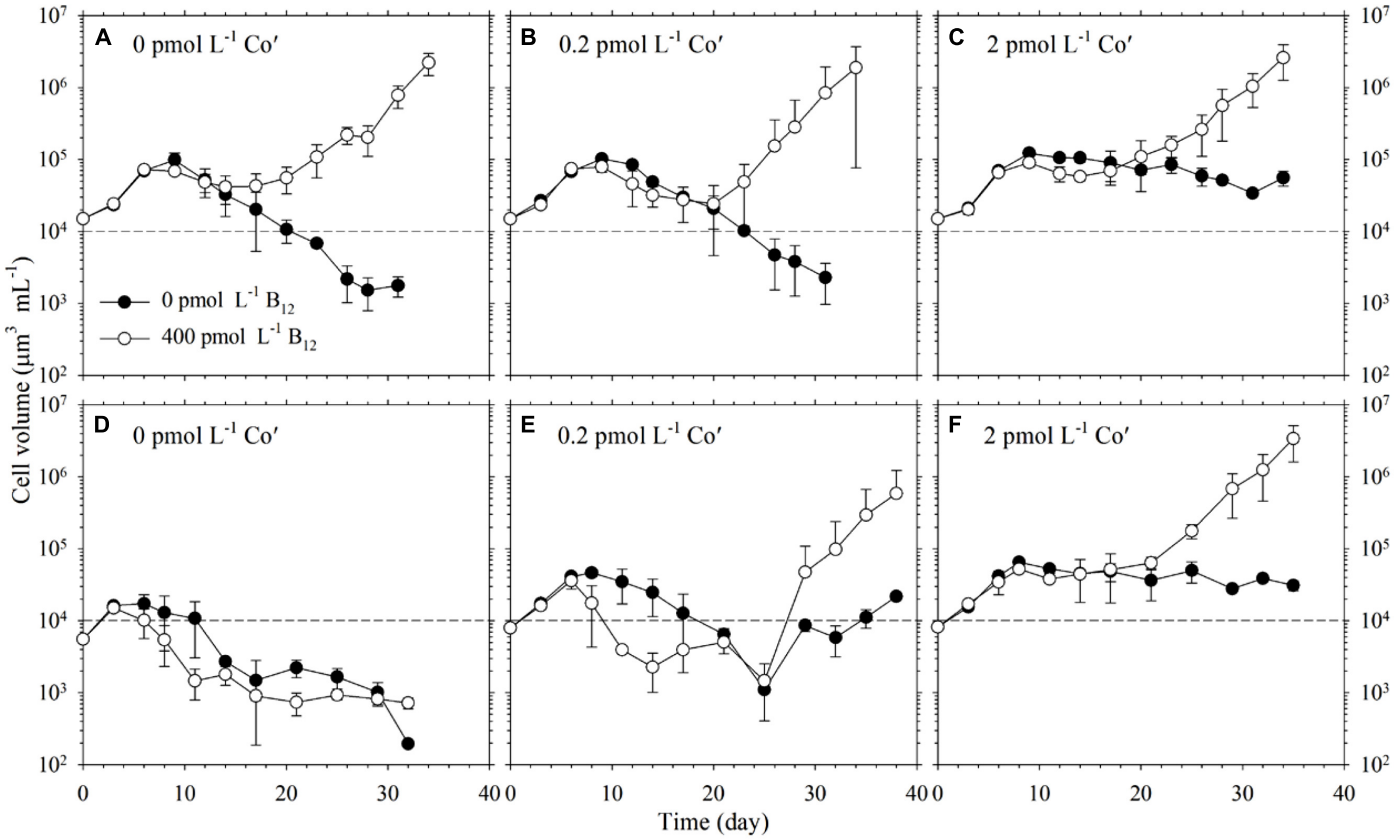
**Growth of *Trichodesmium* in varying Co′ and B_12_ concentrations. (A–C)** Top panels show growth curves of the first transfer of cells in different Co and B_12_ conditions. All treatments were inoculated using cells grown in a medium with 20 pmol L^-1^ Co′ and 400 pmol L^-1^ B_12_ for more than 10 generations. **(D–F)** Bottom panels show growth curves of the second transfer, which were inoculated from cells in the first transfer grown in respective Co treatments with 400 pmol L^-1^ B_12_. Values represent mean and error bars represent SD of triplicate culture bottles. Negative bars for several points in **(B,E)** were long and were not plotted as a result of the log-scale used in the *Y*-axis.

We then varied both Co and B_12_ availabilities to study whether these would interact and affect *Trichodesmium* growth. To achieve this, we conducted parallel experiments with different Co′ and B_12_ concentrations, where Co′ concentrations were 0, 0.2, and 2 pmol L^-1^ and B_12_ concentration was either 0 or 400 pmol L^-1^ for a total of six different treatments (**Figure 2**). The cells used as inoculum for first transfer were previously grown in a medium with 20 pmol L^-1^ Co′ and 400 pmol L^-1^ B_12_ for about 10 generations. We opted to monitor growth upon first transfer because prior acclimatization experiments we conducted resulted in low density or cell death in cultures with low Co′ concentrations. Thus, we decided to monitor growth upon transfer to low Co′ concentrations and observe the phytoplankton response. All of the different treatments increased in biomass upon transfer but either a noticeable decline or only a slight change in biomass was observed after about 8 days (**Figures 2A–C**). The treatments with B_12_ then eventually increased in biomass reaching cell densities needed for the second transfer. For the treatments without B_12_, only cultures with 2 pmol L^-1^ Co′ were able to attain a stationary biomass that reached a maximum of 10^5^ μm^3^ mL^-1^. The cells in the first transfer from different Co treatments and with B_12_ added were used to inoculate the second transfer for corresponding Co treatments (**Figures 2D–F**). In the 0 pmol L^-1^ Co′ treatments, cultures in both B_12_ conditions declined in biomass until these reached cellular volumes that were less than 10^3^ μm^3^ mL^-1^ (**Figure 2D**). For cultures with 0.2 pmol L^-1^ Co′ added, the biomass in both B_12_ treatments initially declined and then eventually increased after about 25 days of incubation (**Figure 2E**). The growth curves of cultures with 2 pmol L^-1^ Co′ (**Figure 2F**) were similar to corresponding treatments in the first transfer. In this Co′ concentration, the treatments with B_12_ attained higher cell density than in the treatments without B_12_.

**FIGURE 3:**
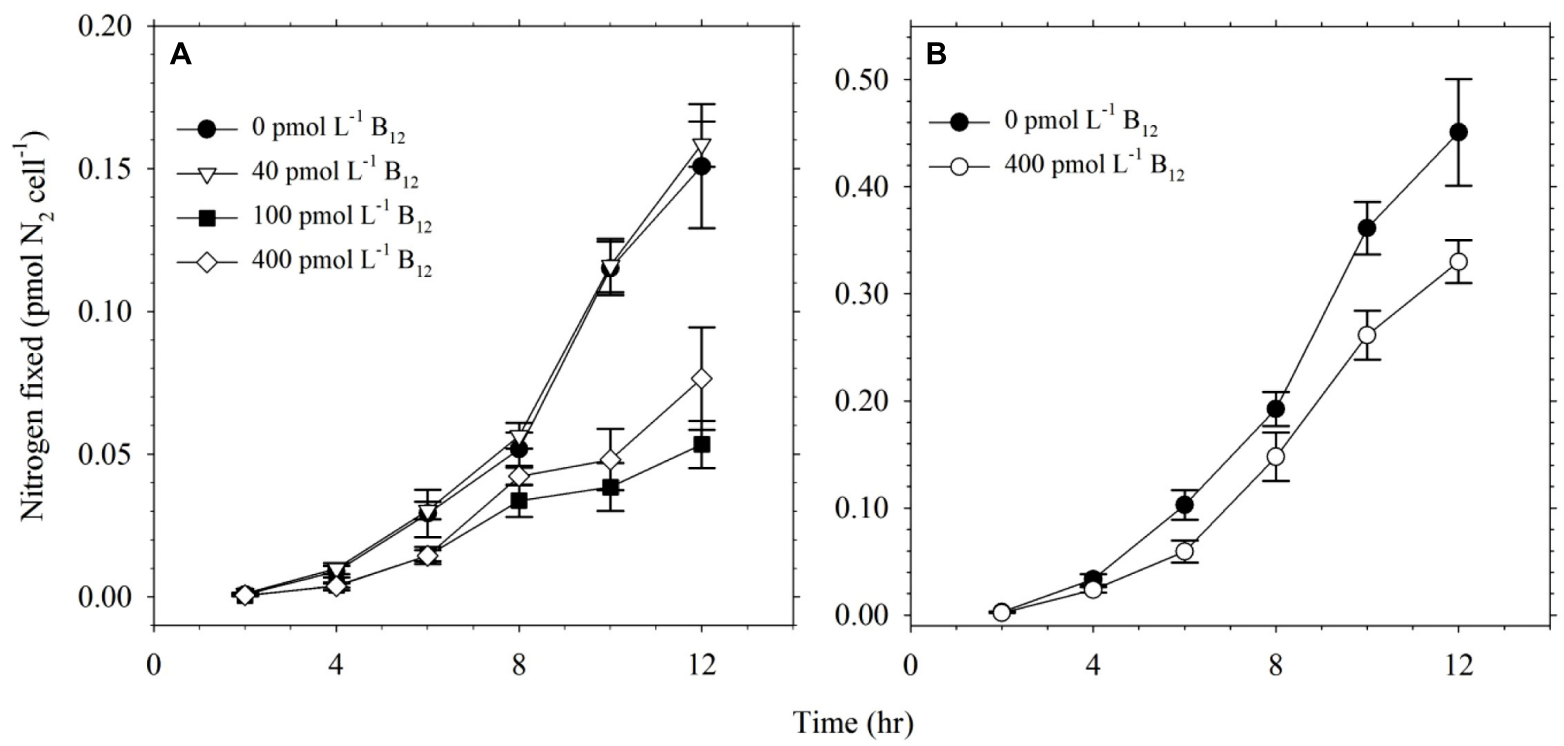
**Nitrogen fixed by *Trichodesmium* grown in culture media with 20 pmol L^-1^ Co′ and different B_12_ concentrations. (A)** Nitrogen levels fixed by *Trichodesmium* in media with 0, 40, 100, and 400 pmol L^-1^ B_12_. **(B)** Nitrogen levels fixed by *Trichodesmium* in media with 0 and 400 pmol L^-1^ B_12_ during the validation experiment. The higher values observed in the validation experiment was due to use of commercially available high purity acetylene as opposed to laboratory-prepared acetylene used in the original experiment. Nitrogen fixation was evaluated using acetylene reduction assay carried out during the 12 h light period while cells were in exponential phase of growth. Values represent mean and error bars represent SD of triplicate culture bottles.

The effect of B_12_ concentration on nitrogen fixation by *Trichodesmium* was also studied under conditions of sufficient Co availability. Our results show that B_12_ concentration influenced the nitrogen fixation by *Trichodesmium* (**Figure 3A**). Cultures without B_12_ and lower B_12_, i.e., 0 and 40 pmol L^-1^, fixed higher amount of N_2_ compared to cultures with higher B_12_ concentrations, i.e., 100 and 400 pmol L^-1^. This trend was also observed in the validation experiment carried out focusing only on treatments with 0 and 400 pmol L^-1^ B_12_ (**Figure 3B**). Values for nitrogen fixed by *Trichodesmium* during the validation experiment were higher than values determined in the first experiment because of different purity of acetylene used in the assays. High purity acetylene that was obtained commercially was used in the validation experiment while laboratory-prepared acetylene was used in the first experiment. The trends observed in both experiments indicate that B_12_ availability influences the nitrogen fixing capacity of this phytoplankton.

## Discussion

*Trichodesmium* is an important diazotroph but its Co and B_12_ requirements and their possible interaction have not been reported; thus we deemed it imperative to understand this link. In this study, we observed comparable growth rates and biomass of *Trichodesmium* in treatments with 20 pmol L^-1^ Co′ and varying B_12_ concentrations (**Figure 1**). This inorganic Co concentration is similar to the labile dissolved Co concentration (17.1 pmol L^-1^) that Panzeca et al. (2008) observed in one of their study areas in the North Atlantic Ocean. Dissolved Co levels in marine waters occur in low pmol L^-1^ levels reaching up to 1900 pmol L^-1^ in areas where there are anthropogenic sources, but Co is bound by strong ligands rendering it unavailable (Saito and Moffett, 2001; Panzeca et al., 2009). The intracellular Co quotas provide useful information to evaluate Co requirement and uptake by *Trichodesmium* (**Table 1**). The average Co uptake rates were estimated by multiplying specific growth rate with cellular concentration. In growth conditions with 20 pmol L^-1^ Co′, we observed that P-normalized Co quotas were comparable among different B_12_ treatments. These Co quotas were three orders of magnitude lower than Fe quotas measured in this study, which generally ranged from 15 to 20 mmol mol^-1^ P. The intracellular Co concentrations in culture medium volume were within 9 ± 2 pmol L^-1^ among all treatments and account for about half of Co′ concentrations initially added to the culture medium. The quotas normalized to cellular volume, ranged from 4.6 to 7.3 nmol L^-1^, indicating a concentration factor in the range 500–700-fold. These quotas support the notion that 20 pmol L^-1^ Co′ was sufficient for *Trichodesmium* to reach maximum growth under the culture conditions.

It has long been posited that Co may indirectly control phytoplankton growth and influence community structure because it is inherently needed in B_12_ synthesis (Panzeca et al., 2009; Bertrand et al., 2012; Sañudo-Wilhelmy et al., 2012). Results from our experiments denoted that B_12_ supply was not a major factor for *Trichodesmium* growth when ample Co was supplied although the growth rate observed in the 0 pmol L^-1^ B_12_ treatment were slightly different from the rates observed in the higher B_12_ treatments (*P* = 0.0213, **Table 1**). Our subsequent results, however, show that under Co′ values equal to or lower than 2 pmol L^-1^, B_12_ availability plays an important role in relieving stress due to Co limitation and it may augment the low Co supply (**Figure 2**). Growth of *Trichodesmium* in culture media with limiting Co′ values reveals that the Co conditions are not suitable to sustain growth rates that were observed in cultures with 20 pmol L^-1^ Co′. With sufficient Co, *Trichodesmium* achieved the highest biomass after only 15 days of incubation (**Figure 1**). At low Co conditions, however, some treatments only reached the equivalent biomass after more than 30 days of incubation (**Figure 2**). The first transfer with varying Co and B_12_ concentrations, inoculated using cells grown in media with 20 pmol L^-1^ Co′ and 400 pmol L^-1^ B_12_, show that Co′ values equal to or lower than 2 pmol L^-1^ were insufficient to support higher phytoplankton biomass if B_12_ was not added (**Figures 2A–C**). We monitored the cell growth starting from first transfer because prior experiments intended for acclimatization to varying Co and B_12_ conditions resulted in cell death, which is also evident in some treatments shown in **Figure 2**. The second transfer of *Trichodesmium*, inoculated from respective Co treatments and with 400 pmol L^-1^ B_12_ in the first transfer, revealed similar trends as in the first transfer (**Figures 2D–F**). This similarity indicates that the observed response may be attributed to the Co and B_12_ concentrations used. The stationary values or declining biomass observed in the treatments without B_12_ show that B_12_ supply was critical for *Trichodesmium* to grow at these Co′ concentrations. This was also directly validated by growth of the phytoplankton in respective Co treatments with B_12_. We speculate that B_12_ transformation, either intracellularly or extracellularly, provides extra bioavailable Co for *Trichodesmium* to recover its growth. Previous studies on *Crocosphaera watsonii* and *Cyanothece* show that these diazotrophs are able to recycle intracellular trace metals in molecules under stressed condition (e.g., Saito et al., 2011; Jacq et al., 2014; Rabouille et al., 2014). There is a possibility that *Trichodesmium* may be able to reuse Co by breaking down B_12_ to compensate for the low Co availability in the culture medium.

Our results clearly show that *Trichodesmium* has an absolute Co requirement and in Co-limiting conditions, this requirement can be partly met with sufficient B_12_ supply. So far, Co has been mainly studied in its role associated to the corrin ring of B_12_ but there are other Co-containing proteins like methionine aminopeptidase, prolidase, nitrile hydratase, glucose isomerase, methylmalonyl-CoA carboxytransferase, aldehyde decarbonylase, lysine-2,3-aminomutase, and bromoperoxidase (Kobayashi and Shimizu, 1999). These non-corrin Co-containing enzymes have been isolated and characterized but further research is needed to fully understand their biological functions. Aside from its role in the enzymes mentioned, Co may also substitute for Zn in carbonic anhydrase under Zn-limited conditions for some eukaryotic phytoplankton (Sunda and Huntsman, 1995). Moreover, a Co-containing alkaline phosphatase has been identified in bacteria isolated from a hot spring although alkaline phosphatase normally contains Zn as a metal cofactor (Gong et al., 2005). The role of Co in phosphorus acquisition has been demonstrated in a culture study by Jakuba et al. (2008) using the coccolithopore *E. huxleyi* and they found that low Co treatments resulted in low alkaline phosphatase activity. All treatments in our culture study were supplemented with 12.5 pmol L^-1^ Zn′ but the observed trends suggest that Co was not replaced with Zn. The existence of various enzymes that require Co warrants further studies to comprehensively understand biochemical processes involving Co and identify which of these may be important in *Trichodesmium*.

B_12_ and other B-vitamins may come from biological production and other sources including allochthonous sources, such as influxes from rivers and streams, and regenerated vitamins from phytoplankton cell death or lysis (Gobler et al., 2007). These sources lead to B_12_ concentrations that are higher in coastal waters than in the open ocean and which generally reflects abundance of total Co concentration (Panzeca et al., 2008). Low B_12_ concentrations are typical in the tropical and subtropical oceans characterized by intense light and high temperatures explaining the relatively short half-life of B_12_ in the field (Carlucci et al., 1969). The highest B_12_ concentration we used in this work (400 pmol L^-1^ B_12_) is approximately equivalent to the suggested B_12_ level in YBC-II medium widely used to cultivate *Trichodesmium* in the laboratory. *Trichodesmium* has been shown to harbor various bacteria especially when present in colonies (Sheridan et al., 2002; Roe et al., 2012). The strain used in this work is non-axenic, thus it may contain bacteria taken along with it since it was isolated and subsequently cultivated in the laboratory. Bacteria and other organisms associated with *Trichodesmium* are both particle attached or free-living and were found to be specific, indicating a certain degree of relationship between host and associated bacteria (Sheridan et al., 2002). B_12_ synthesis is a feature unique to some prokaryotic organisms and some of the associated bacteria in *Trichodesmium* may be capable of B_12_ synthesis, thus this association is possibly related to B_12_ cycling. The genome of *Trichodesmium* shows that it contains genes necessary for B_12_ synthesis (Sañudo-Wilhelmy et al., 2014). Therefore, it is imperative to take into account both diazotroph and associated organisms in *Trichodesmium* cultures when considering the results we observed. Our results show that growth of *Trichodesmium* in media with sufficient Co was independent of B_12_ concentration (**Figure 1**). B_12_ supply, however, was crucial for the growth of the diazotroph when Co was insufficient (**Figure 2**). These results demonstrate that *Trichodesmium* in cultures may either synthesize the B_12_ it requires or rely on associated bacteria to produce B_12_ for optimal growth. Whether it is *Trichodesmium* or its associated bacteria producing B_12_, the process certainly merits further study because this represents an additional source of the important organic growth factor in otherwise B_12_-limited environments.

B_12_ is a nitrogen-rich molecule with multiple uses in important biochemical processes. Thus we studied whether its availability will affect the nitrogen fixing capacity of *Trichodesmium* or not. We carefully prepared culture media for cultivating this diazotroph to ensure that nitrate concentration was kept at a minimum. The nitrate concentrations in the culture media were close to the detection limit, which is around 0.05 μmol L^-1^. Results from the acetylene reduction assay reveal that this diazotroph fixed more N in conditions without B_12_ or with lower B_12_ concentration (**Figure 3A**). The nitrogen levels fixed by cultures with higher B_12_ concentrations were comparable to values we obtained in our previous work (Rodriguez and Ho, 2014). Trends observed in the validation experiments ascertain our observation that B_12_ availability influences the nitrogen fixing capacity of *Trichodesmium* (**Figure 3B**). Levels of nitrogen fixed were lower in the first experiment due to use of laboratory-prepared acetylene as opposed to commercially available acetylene used in the validation experiment. It has been reported that use of high purity acetylene results in a more sensitive detection (Kitajima et al., 2009), which we have also observed in this work. Although the difference in purity of acetylene produced significant discrepancies in determined values, both experiments produced similar trends showing that lower B_12_ availability result in higher nitrogen fixation by the diazotroph. These results show that B_12_ availability affects nitrogen fixation up to a certain extent and this difference may be of significance in certain oceanic regions where B_12_ is depleted. The higher amounts of nitrogen fixed by cultures grown in low-B_12_ and without-B_12_ treatments indicate that B_12_ concentrations in the open ocean, which typically occurs in the range 0.3–60 pmol L^-1^ (Panzeca et al., 2009; Sañudo-Wilhelmy et al., 2012), may influence nitrogen fixation by this phytoplankton. Although it is tempting to propose that higher nitrogen fixation in treatments with low B_12_ was due to nitrogen requirement for B_12_ synthesis, the presence of 14 N atoms in a B_12_ molecule cannot quantitatively account for the observed difference. The role of B_12_ in nitrogen fixation by *Trichodesmium* requires further in-depth probing to understand its function in this diazotroph.

The observation that low B_12_ concentration enhances nitrogen fixation by *Trichodesmium* entails that it may play a crucial role in the oceanic regions where it thrives. A recent work surveying the B_12_ distributions in the open ocean found that B_12_ is severely depleted in oligotrophic areas forcing most phytoplankton to live under B_12_ deficiency (Sañudo-Wilhelmy et al., 2012). The presence of B_12_ producers in this environment that is also nitrogen-limited may therefore radically change predominant conditions. *Trichodesmium* and its associated bacteria can thrive in oceanic waters that have limited bioavailable nitrogen because the diazotroph can take advantage of the unlimited atmospheric N_2_ resource. Whether it is *Trichodesmium* or its associated bacteria producing B_12_, both can benefit from bioavailable nitrogen produced by the diazotroph. This is an inherent advantage over other B_12_ producers that are limited by bioavailable nitrogen supply as has been shown in the non-diazotrophic species *Synecochoccus* (Bonnet et al., 2010). The colimitation between B_12_ and nitrogen has been noted to impact phytoplankton group composition (Bertrand and Allen, 2012). These advantages further highlight the role of *Trichodesmium* in the dynamics of phytoplankton community structure and in global cycling of nutrients. The availability of Co, however, will dictate the growth and nitrogen fixation of *Trichodesmium*. While we cannot attribute the absolute Co requirement to a specific biochemical demand, it is clear from our results that *Trichodesmium* requires Co for its growth. Because of the importance of *Trichodesmium* in oligotrophic ocean, Co availability can therefore impact primary production and nutrient cycling in these regions.

## Conflict of Interest Statement

The authors declare that the research was conducted in the absence of any commercial or financial relationships that could be construed as a potential conflict of interest.
